# Differential Diagnosis Assessment in Ambulatory Care With an Automated Medical History–Taking Device: Pilot Randomized Controlled Trial

**DOI:** 10.2196/14044

**Published:** 2019-11-04

**Authors:** Adrien Jean-Pierre Schwitzguebel, Clarisse Jeckelmann, Roberto Gavinio, Cécile Levallois, Charles Benaïm, Hervé Spechbach

**Affiliations:** 1 Division of Physical Medicine and Rehabilitation Department of Rheumatology Lausanne University Hospital Lausanne Switzerland; 2 Faculty of Medicine University of Lausanne Lausanne Switzerland; 3 Ambulatory Emergency Care Unit Department of Primary Care Medicine Geneva University Hospitals Geneva Switzerland

**Keywords:** differential diagnosis, decision making, computer-assisted, hospital outpatient clinics, general practitioners, clinical applications software, patient engagement

## Abstract

**Background:**

Automated medical history–taking devices (AMHTDs) are emerging tools with the potential to increase the quality of medical consultations by providing physicians with an exhaustive, high-quality, standardized anamnesis and differential diagnosis.

**Objective:**

This study aimed to assess the effectiveness of an AMHTD to obtain an accurate differential diagnosis in an outpatient service.

**Methods:**

We conducted a pilot randomized controlled trial involving 59 patients presenting to an emergency outpatient unit and suffering from various conditions affecting the limbs, the back, and the chest wall. Resident physicians were randomized into 2 groups, one assisted by the AMHTD and one without access to the device. For each patient, physicians were asked to establish an exhaustive differential diagnosis based on the anamnesis and clinical examination. In the intervention group, residents read the AMHTD report before performing the anamnesis. In both the groups, a senior physician had to establish a differential diagnosis, considered as the gold standard, independent of the resident’s opinion and AMHTD report.

**Results:**

A total of 29 patients were included in the intervention group and 30 in the control group. Differential diagnosis accuracy was higher in the intervention group (mean 75%, SD 26%) than in the control group (mean 59%, SD 31%; *P*=.01). Subgroup analysis showed a between-group difference of 3% (83% [17/21]-80% [14/17]) for low complexity cases (1-2 differential diagnoses possible) in favor of the AMHTD (*P*=.76), 31% (87% [13/15]-56% [18/33]) for intermediate complexity (3 differential diagnoses; *P*=.02), and 24% (63% [34/54]-39% [14/35]) for high complexity (4-5 differential diagnoses; *P*=.08). Physicians in the intervention group (mean 4.3, SD 2) had more years of clinical practice compared with the control group (mean 5.5, SD 2; *P*=.03). Differential diagnosis accuracy was negatively correlated to case complexity (*r*=0.41; *P*=.001) and the residents’ years of practice (*r*=0.04; *P*=.72). The AMHTD was able to determine 73% (SD 30%) of correct differential diagnoses. Patient satisfaction was good (4.3/5), and 26 of 29 patients (90%) considered that they were able to accurately describe their symptomatology. In 8 of 29 cases (28%), residents considered that the AMHTD helped to establish the differential diagnosis.

**Conclusions:**

The AMHTD allowed physicians to make more accurate differential diagnoses, particularly in complex cases. This could be explained not only by the ability of the AMHTD to make the right diagnoses, but also by the exhaustive anamnesis provided.

## Introduction

### Background

In studies performed in the United States on medical errors in primary care medicine, diagnostic errors are the most common [1-3] and the most expensive [4,5], as well as the cause of most malpractice claims [1,4,6]. A prevalence of diagnostic errors in outpatient care of at least 5% has been reported [7]. Despite their importance, diagnostic errors are underemphasized and underidentified [6,8], and the development of novel strategies to improve the accuracy of the initial diagnosis should be a priority.

Interactive computerized interviews completed by patients have several advantages and are shown to be as accurate as classic clinician records. Notably, they permit a significant difference in time taken during the consultation [9], thus demonstrating that the initial triage could be performed in less time [10]. Physicians also receive more data than that from conventional history taking [11-15]. In addition, false positive answers to classic interviews may less likely occur as answers could be optional, thus allowing blank responses [16]. In the waiting room, patients have reported high satisfaction by helping their physician through the completion of interactive computerized interviews [17,18]. The interview is better organized and permits the physician to easily consolidate the anamnesis with supplementary questions, depending on the data provided [16]. Patients are also more likely to reveal sensitive data to a computer than to a physician [19-21]. Finally, the process is an effective strategy to empower patients to be active in their own care (patient engagement) [22,23].

At present, 2 types of interactive computerized interviews exist to facilitate the anamnesis and diagnosis before the consultation, that is, symptom checkers and automated medical history–taking devices (AMHTDs). Recently, 23 symptom checkers were evaluated with standardized vignettes. The correct diagnosis was made in 58% of the cases, and a correct triage was performed in 80% [24], which can be considered as insufficient. Another solution includes an AMHTD based on a single symptom or localization [17]. This type of system can be useful and accurate, provided that the clinical presentation is typical, for example, a patient presenting with calf pain after strenuous exercise and a potential sciatica.

### Objectives

The primary aim of this pilot study was to investigate whether the DIAANA AMHTD allowed physicians to establish a more accurate DD, with the DD of a senior physician considered as the gold standard. Secondary aims were to assess the accuracy of the DD list established by the AMHTD, identify factors that might influence the usefulness of the AMHTD, and evaluate physician and patient satisfaction with its use.

We tested a novel AMHTD, named *DIAANA* (DIAgnosis & ANAmnesis; created by Logic-based Medicine Sàrl), to help the physician to establish the differential diagnosis (DD) more accurately, based on broad possibilities of disease or trauma localization, triggering factors, and symptoms. The physician can therefore begin his consultation with an exhaustive anamnesis summary including a more precise localization and nature of symptoms as well as a high-sensitivity DD list with corresponding triggering factors for each diagnosis. We consider that this tool could help the physician in his/her diagnostic reasoning and to perform tasks more efficiently, without being substituted by the AMHTD.

## Methods

### Study Design

We conducted a pilot, single-center, unblinded, 1:1 parallel-group, randomized efficacy trial. No follow-up was necessary. There were no changes in the protocol after trial commencement. The study protocol was optimized and approved by an independent expert methodologist. It was not registered as it was considered to be a pilot phase. Given that recruitment began just after the approval, it would therefore have not been relevant to register the study after the beginning of the recruitment. The protocol was approved by the Medical Ethics Committee of Geneva University Hospitals (Geneva, Switzerland; REQ-2017-00878). No bugs were fixed during the trial. As this was a purely observational study without identifiable side effects or negative consequences for patients, only oral informed consent was obtained, supported by a brief written description of the project. Consolidated Standards of Reporting Trials of Electronic and Mobile Health Applications and online TeleHealth V 1.6 (see Multimedia Appendix 1) was used to improve and standardize the quality of this paper [25].

### Patient Population

From May to September 2018, we prospectively enrolled adult patients presenting to the emergency outpatient unit of our institution and suffering from symptoms covered by the AMHTD. Symptoms were localized to the superior member (apart from the hand, as the device had not yet been programmed to take related conditions into consideration), the trunk, and the inferior member, with the exception of strictly dermatologic concerns and toes and inversion ankle trauma as the diagnosis is generally obvious. We excluded patients with a medical situation considered as urgent and unable to complete the digitalized AMHTD (sight problems, advanced age, and non-French-speaking). Patients were enrolled only when one of the senior physicians in charge of the project (CL, TW, RG, MB, and HS) and one of the coordinators (CJ and BV) were available.

### Randomization and Recruitment

At the beginning of the study, 18 residents of the emergency outpatient unit were stratified, and 1:1 matched by their years of clinical experience (orthopedics, rheumatology, and physical medicine counted twice) and then randomized. When a patient was allocated to a resident physician using the emergency software system, the coordinating researcher evaluated the patient’s potential eligibility. The senior physician then confirmed the patient’s eligibility and applied the exclusion criteria. Depending on the resident physician’s allocation, the patient was included in either the intervention or the control group. In each group, the recruitment was blocked after the inclusion of 30 patients.

### DIAANA Tool Presentation

The DIAANA AMHTD functions as follows: On the basis of an interactive questionnaire completed by the patient before the consultation, which includes 269 questions (mainly multiple choice), it performs an exhaustive anamnesis focused on the problem and proposes a panel of DDs with a high sensitivity, selected on a panel of 126 diagnostic entities. The artificial reasoning system of DIAANA mimics how a specialist physician would reason to establish a DD. The information transmitted is in an easy-to-use form for the physician that includes a summary of the anamnesis centered on relevant elements from the questionnaire and a list of possible diagnoses with their emergency level, potential contributing factors, and first-line management proposals. Multimedia Appendix 2 illustrates an example of a patient suffering from deep vein thrombosis that was initially confounded with a tennis leg. More detailed information is available on the AMHTD’s website [26].

### DIAANA Tool Development

For 3 years, AS was involved in the development of the AMHTD, taking into consideration all aspects of the diagnosis and management of orthopedic, rheumatologic, vascular, neuropathic, and sports-related medical conditions, with the help of a few sources [27-29] as well as peer advice.

The system was built with triggering conditions that are turned on when the patient selects a specific answer. The triggering condition will then call up new questions and diagnostic entities. As an example, if the patient clicks *leg* on the general localization, the trigger *leg* is turned on, and a more specific question about the leg localization appears (see Multimedia Appendix 2). AS built a first draft of DIANNA including the principal questions of a proper musculoskeletal anamnesis. Then, he considered the 126 selected diagnosis entities in more depth and added more specific questions for each diagnosis step by step. The accuracy of DIANNA depends, therefore, on the accuracy of the patient’s answer as well as the exhaustivity of the questions and diagnostic entities. As an example, if the correct localization (eg, *ankle*) is not selected, specific questions (eg, trauma in external rotation) and a specific diagnosis (eg, syndesmosis sprain) will not be triggered and thus be missing in the DIANNA summary.

Hundreds of episodes of testing with healthy volunteers, medical students, and patients were performed during the development process, and the formulation of questions, triggering conditions, and the DIANNA summary were adjusted according to feedback from users. A final development phase was conducted with the feedback of 20 patients presenting to the emergency outpatient unit, and the first version of the digital content of the tool was then frozen for the pilot study. This frozen version remains available upon request to the corresponding author.

### Intervention

In the intervention group, patients in the AMHTD group were asked to complete a digital form on a touch pad by the coordinator (and without help) before the medical consultation. The AMHTD summary was then printed and given to the resident physician before the consultation. At the end of the consultation, but before consulting the complementary medical examination results (radiographs and blood laboratory results), the resident physician established his/her DD on the diagnosis list (see Multimedia Appendix 3**)** on a touch pad, without the help of the research coordinator**.** In parallel, the senior physician established the gold standard DD on the same list. In the control group, the resident physician established his DD on the diagnosis list at the end of the consultation, but before consulting the complementary medical examinations. The senior physician followed the same procedure. For ethical reasons, the use of the AMHTD had no influence on patient care as the clinical management was fully decided upon by the senior physician who had no access to the summary generated.

### Outcomes

The primary outcome was the percentage of correct DDs established by the resident physician compared with the senior physician. Secondary outcomes included (1) the percentage of correct AMHTD DDs and the percentage of correct AMHTD DDs followed/not followed by the resident, as well as the percentage of incorrect AMHTD DDs followed by the resident and the number of incorrect AMHTD DDs; (2) overall patient satisfaction on the understandability of AMHTD questions (1-5 Likert scale), ability to describe symptoms accurately (percentage), and respect of the patient’s wish to use the AMHTD at home and to keep the generated summary (percentages); (3) resident’s feedback on the wish to obtain the integrality of the AMHTD summary (percentage), whether the AMHTD found DDs that would have been omitted otherwise (percentage), and if the use of the device saved time (1-5 Likert scale); and (4) the percentage of correct DDs depending on case complexity, defined as the number of DDs present in the gold standard DD (1-2 DDs=low complexity; 3 DDs=intermediate complexity; and 4-5 DDs=high complexity). The stratification for the case complexity definition used has never been published. The rationale was to highlight that the AMHTD was built and conceived to help the physician when the diagnosis might be confusing or in the case of a complex situation. Indeed, it would not be relevant to ask the patient to provide a complete anamnesis if the physician can complete it in 2 min for a problem such as benign soft tissue trauma.

### Statistical Analyses

A sample size of 30 patients per group was chosen as recommended for pilot studies to achieve an appropriate level of statistical power [30]. It corresponds to the detection of a potential difference of 21% between groups for a power of 80% and an alpha significance level of 5%. Descriptive statistics were used to describe baseline characteristics. Differences between groups in the intention-to-treat analysis were evaluated using Student *t* test or the Wilcoxon rank-sum test, when appropriate. Analysis of covariance was performed considering the covariables of interest (primary outcome, case complexity, and resident’s years of experience) with a *P* value <.20 considered as significant in univariate analysis. *P* values <.05 were considered as statistically significant. All analyses were performed using R v3.4.2 Portable (Free Software Foundation Inc).

## Results

### Population

**Figure 1:**
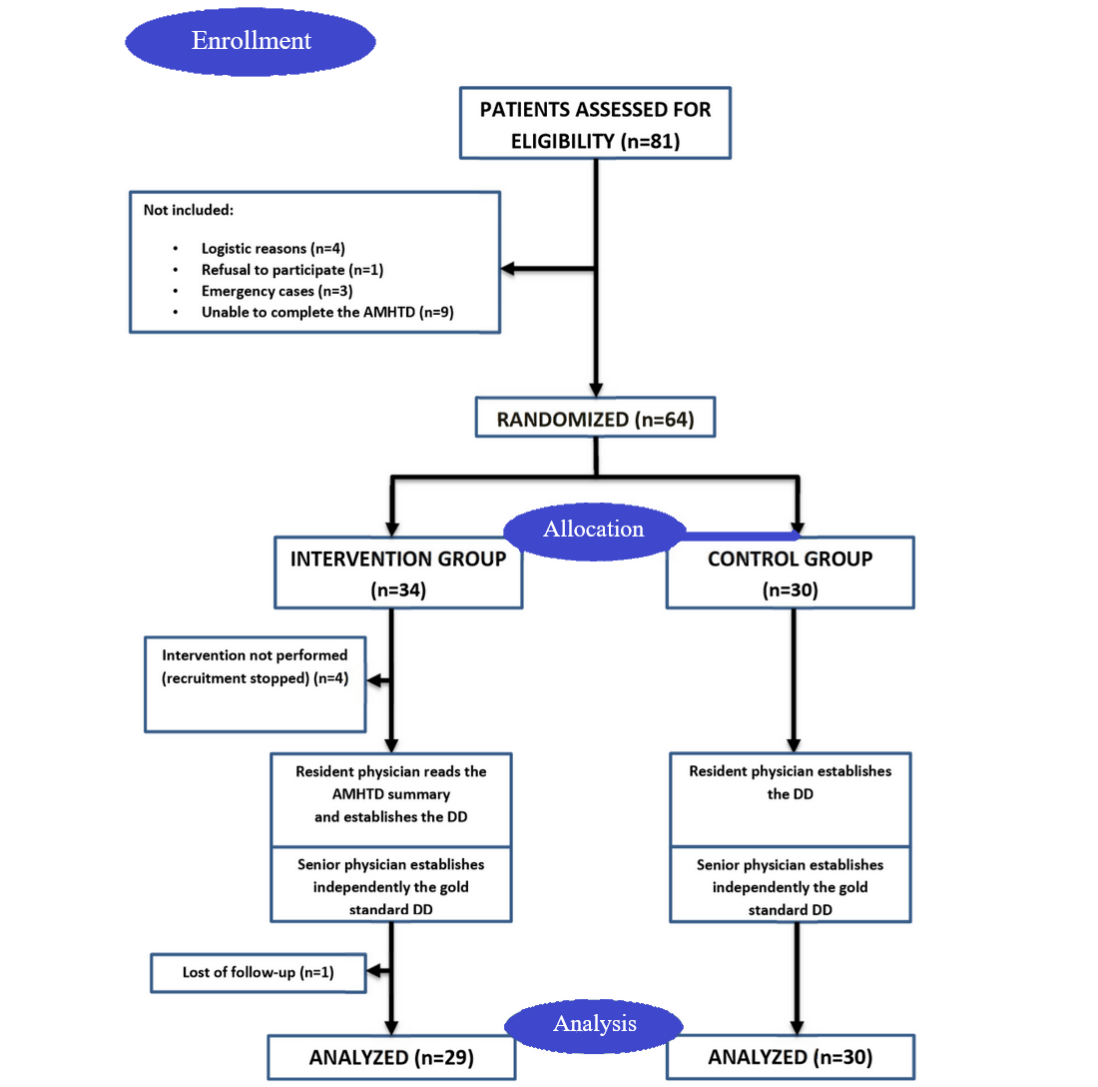
Study flow chart. AMHTD: automated medical history–taking device; DD: differential diagnosis.

Of the 81 patients screened, 64 were randomized and allocated to residents (Figure 1). Among the randomized patients, 4 allocated to the intervention group were not included as 30 patients were already included in the intervention group; 1 patient was lost to follow-up. In the final analysis, 29 patients were included in the intervention group and 30 in the control group. Preintervention patient demographics, case complexity, and initial complaint/s did not differ between the groups (Table 1). Residents in the control group had more years of practice (*P*=.03).

**Table 1:** Baseline characteristics.

Baseline characteristics		AMHTD^a^ (n=29)	Control group (n=30)	*P* value
**Age (years)**				
	Mean (SD)	38 (14)	42.1 (16)	.29
	Range	17-66	19-75	.29
Male gender, n (%)		23 (79)	22 (73)	.82
**Physician’s practice (years)**				
	Mean (SD)	4.3 (2)	5.5 (2)	.03
	Range	3-8	3-8	.03
**Case complexity (number of differential diagnoses to find)**				
	Mean (SD)	3.1 (1)	2.9 (1)	.60
	Range	1-5	1-5	.60
**Initial complaint, n (%)**				
	Elbow pain	1 (3)	1 (3)	>.99
	Shoulder pain and trauma	3 (10)	2 (7)	.97
	Back pain and trauma	5 (17)	7 (23)	.80
	Pelvic pain	2 (7)	0 (0)	.46
	Knee pain and trauma	8 (28)	6 (20)	.70
	Ankle trauma	4 (14)	6 (20)	.77
	Foot trauma	2 (7)	2 (7)	>.99
	Soft tissue trauma and swelling	4 (14)	5 (17)	>.99

^a^AMHTD: automated medical history–taking device.

### Analysis of Accuracy of Differential Diagnosis

In the univariate analysis, the percentage of correct DDs was (1) higher in the intervention group (mean 75% [SD 26%] vs mean 59% [SD 31%], respectively; *P*=.03); (2) negatively correlated to case complexity (*r*=0.41; *P*=.001); and (3) negatively correlated to residents’ years of practice (*r*=0.04; *P*=.72). The *P* value of the analysis of covariance model, including the percentage of DDs found and case complexity was .01. Considering case complexity, we observed between-group differences in favor of the AMHTD of 3% (83% [17/21]-80% [14/17]) for low-complexity cases, 31% (87% [13/15]-56% [18/33]) for intermediate-complexity cases, and 24% (63% [34/54]-39% [14/35]) for high-complexity cases (Table 2). The type of DD made by the senior physician, depending on the case complexity, is presented in the Multimedia Appendix 4.

By comparison, the AMHTD was able to find 73% (SD 30%) of correct DDs for the whole cohort: 91% (SD 20%) for low-complexity cases; 67% (SD 24%) for moderate-complexity cases; and 58% (SD 32%) for high-complexity cases (see Multimedia Appendix 5). The AMHTD also proposed 5(SD 4) incorrect diagnostic proposals. Residents did not list 10% (SD 19%) of the correct DDs proposed by the AMHTD and listed 21% (SD 51%) of incorrect DDs.

**Table 2:** Percentage of correct differential diagnoses per group.

DD^a^ studied	AMHTD^b^ (n=29)		Control group (n=30)		Univariate analysis *P* value	Multivariate analysis *P* value
	Mean (SD)	Range	Mean (SD)	Range		
DD accuracy	75 (26)	25-100	59 (31)	0-100	.03	<.001
Low complexity (1-2 DDs to find)	83 (25)	50-100	80 (26)	50-100	.76	—^c^
Moderate complexity (3 DDs to find)	87 (18)	67-100	56 (26)	0-100	.02	—
High complexity (4-5 DDs to find)	63 (25)	25-100	39 (29)	0-80	.08	—

^a^DD: differential diagnosis.

^b^AMHTD: automated medical history–taking device.

^c^Not applicable.

### Users Satisfaction

Patient satisfaction was good regarding overall satisfaction with questions and their understandability, and 26 of 29 (90%) patients considered that they were able to accurately describe their symptoms. Of note, 14 of 29 (48%) patients wished to use the AMHTD at home, and 20 of 29 (69%) resident physicians wished to obtain the full report of the AMHTD. Although 8 of 29 (28%) residents considered that the device helped to establish the DD, they estimated overall that the AMHTD was neither time-saving nor time-wasting (see Multimedia Appendix 6).

## Discussion

### Principal Findings

Our results confirmed that the AMHTD significantly allowed the physician to establish a more exhaustive DD (from 59% to 75%). This effect was more important in moderate-complexity (from 56% to 87%) and high-complexity (from 39% to 63%) cases. Of note, the diagnostic list established by the AMHTD was not as accurate as expected (73%, 66/90) and was more precise for low-complexity cases. Overall patient satisfaction (4.3/5) was good, including the ability to accurately describe the presented symptomatology (90%, 26/29). Thus, our results were in agreement with the main factors that guarantee the success of electronic health (eHealth) [31], that is, an improved diagnosis and clinical management, as well as patient-centered care. Our panel of patients presenting to the outpatient unit had common pathologies and was managed by residents at the end of their training. These conditions are common in outpatient services in Switzerland, and our results should be applicable to other hospitals in the country.

### Limitations

Our study has some limitations. First, it was an unblinded pilot study with a limited sample size in 1 care center. Therefore, we did not anticipate statistically significant results and did not register our protocol following ethics committee approval. Second, our groups were not balanced as resident physicians in the control group had more years of practice, thus leading to a potential selection bias that could have induced an overestimation of the ability to find a correct DD in the control group. Therefore, the positive effect of 16% (75% [68/90]-59% [50/85]) on the accuracy of the DD might be underestimated. Third, even though our senior physicians were experts in the fields of orthopedics and emergency medicine, the gold standard DD might be flawed, especially in more complex cases. This may be a potential explanation for the observed poorer accuracy of the AMHTD DDs in complex cases. Finally, our AMHTD is still under development, and the reliability of patient responses may be suboptimal, especially because of the absence of images to help in patient symptom localization. This could potentially lead to a degree of uncertainty related to the summary generated. Concerning the DIANNA tool digital content, even if we are fully satisfied with the anamnesis summary, the list of diagnoses might lack accuracy.

### Interpretation and Comparison With Prior Research

At present, artificial intelligence systems are still unable to replace physicians for the establishment of a correct DD [31]. Despite this, artificial intelligence allows to complement the work of the physician [32] and even establish an accurate list of problems [33] as shown recently with IBM Watson. The physician’s ability to establish a DD can be improved by providing a case summary and a list of possible diagnoses [32,34]. In contrast with other existing digital systems designed to work hand-to-hand with the physician, such as Ada (Ada Health GmbH), K (K Health), and the Mayo Clinic Symptom Checker (Mayo Clinic), DIAANA is focused on the anamnesis rather than the diagnosis, and highly specialized in injury/disease of the musculoskeletal system. To the best of our knowledge, these abovementioned systems have not been challenged in randomized trials. In addition, we were unable to find any relevant literature concerning other similar systems in the field of general medicine or orthopedics. For instance, in the field of psychiatry, a self-report tool allowed the physician to perform a more accurate diagnosis [35]. Similarly, in acute pediatric assessment, it was shown that junior physicians were able to significantly improve the quality of their diagnostic workup and reduce diagnostic omission errors with the use of a Web-based diagnostic reminder system [36]. These observations are concordant with our results as we showed that it was possible to significantly improve the quality of the DD by providing the physician with an exhaustive anamnesis summary and a list of possible DDs. However, in our study, whether the physician was helped by the exhaustive anamnesis summary or by the DD panel remains open. Both may be useful, although we would suggest that the medical history summary may be superior as the DD panel was not as accurate as expected. Indeed, the DD accuracy of the AMHTD alone (73%, 66/90) was slightly superior to the resident physician in the control group (59%, 55/85), but not superior to the resident physician aided by the AMHTD (75%, 68/90). The reliability of the AMHTD DD without the interpretation of the physician is, therefore, not sufficient. On the other hand, the physician may have underestimated the AMHTD DD reliability, as 10% (9/90) of diagnoses were omitted by residents, but suggested by the AMHTD. This means that if the physician had systematically followed the suggestions of the AMHTD, he/she would have found 85% (78/90) of correct DDs instead of 75% (68/90). The physician should be also aware that the correct diagnosis may be absent on the diagnosis list and, in this case, he/she should not waste energy and resources by trying to explore the entire diagnosis list in depth.

The AMHTD presented was conceptualized as a consultation complement for the physician, and not as a substitute. Physician-informatics partnership is the cornerstone of quality of care improvement, not only because it preserves human relationships [31,37], but also because it is the only condition under which diagnostic assistance has been proven to date. In addition to the existing solutions presented above, it has been shown that patients with unresolved medical issues who submitted their cases on the Web to a panel of specialized case-solvers estimated being helped in their diagnosis process in 60% of the cases [38]. We used the DD as a primary outcome rather than the finally retained diagnosis. Even if only the final diagnosis makes clinical sense, it is well known that only an exhaustive DD can lead to a correct diagnosis with any certainty in medical practice. Using the DD as a primary outcome allowed to increase the effect size because the success rate in establishing a DD is poorer than finding the correct diagnosis. Moreover, to identify situations where a rare but serious diagnosis is missed, thousands of patients should be included if the primary outcome was to be considered as the final diagnosis.

The use of eHealth devices for training purposes is on the rise, as reflected in the increasing use of anamnesis and diagnostic supporting tools used by medical students [39]. We consider that our AMHTD presents ideal characteristics for the training of resident physicians by providing an exhaustive anamnesis and a list of DDs with their degree of emergency and associated factors, as well as initial management guidance. Moreover, the device could be used as a tool for asynchronous teleconsultation.

Workload and workflow disruption are recognized as negative factors influencing the outcome of eHealth interventions [31]. We hypothesized that the exhaustive information collected by the AMHTD would allow the physicians to gain some time. Surprisingly, our physicians estimated that the AMHTD was neither time-saving nor time-wasting. Unfortunately, it was not possible to differentiate the potential time gain for clinical evaluation and reasoning from the time associated with the study itself, for example, contact with the coordinating researcher or waiting for the AMHTD summary to be generated. It is also possible that in low-complexity cases, where the medical history is easily performed, the AMHTD becomes time-consuming. We were unable to measure objectively the consultation time, which may be fragmented when physicians are managing more than one patient at the same time. Completion of the AMHTD form takes some time for patients (20 min in our experience). However, as evidenced by the high satisfaction rate, patients are generally happy to take the necessary time to complete the form. In our study, patients completed the AMHTD form when the waiting time was estimated to be greater than 20 min before the start of the consultation.

Overall, patient satisfaction was good. Of 29 patients, 12 (41%) expressed willingness to keep the AMHTD at home, thus emphasizing the subjective importance for the patients to keep their medical folder and the eHealth tool. We did not provide patients with the AMHTD summary because of the necessity to remain noninterventional in the context of the study for ethical purposes and to avoid causing anxiety to patients when reading highly sensitive DDs. A minority of residents (8/29, 28%) considered the AMHTD as meaningful, and this might reflect the lack of usefulness of the AMHTD for low-complexity cases. Interestingly, 69% (20/29) of physicians wished to obtain the entire AMHTD form, thus potentially highlighting the need to obtain the most accurate and least transformed information as possible, even to the detriment of their time. This contrasts with our initial point of view that the AMHTD summary was sufficient, and the full form would lead to time loss for the physician.

### Conclusions

The tested musculoskeletal-focused AMHTD allowed physicians to make a more accurate DD, particularly for complex cases. This could be explained not only by the ability of the AMHTD to propose the right diagnosis but also by the exhaustive anamnesis provided. Patients and physicians expressed overall satisfaction with the process. On the basis of these pilot study results, further research will aim to assess and clarify the following points: confirmation of the findings and a fine-tuned assessment of the accuracy of the established DD, depending on complexity; objective measurement of consultation time; and an evaluation of the physicians’ learning curve, both in terms of the accuracy of the DD and duration of the consultation.

## Appendix

Multimedia Appendix 1 CONSORT‐EHEALTH checklist (V 1.6.1).

Multimedia Appendix 2 Example of a patient complaining of muscle cramp.

Multimedia Appendix 3 Differential diagnosis list.

Multimedia Appendix 4 Types of differential diagnoses selected by the senior physician for each level of complexity.

Multimedia Appendix 5 Differential diagnoses found by the automated medical history–taking device.

Multimedia Appendix 6 Automated medical history–taking device group: patient and resident physician satisfaction.

## Abbreviations

AMHTD: automated medical history–taking device
DD: differential diagnosis
DIAANA: DIAgnosis & ANAmnesis
eHealth: electronic health
