# Supplementary material for: Differential Diagnosis Assessment in Ambulatory Care With an Automated Medical History–Taking Device: Pilot Randomized Controlled Trial
Source: JMIR Med Inform. 2019 Nov 4;7(4):e14044. doi: 10.2196/14044 (PMC6913752; doi:10.2196/14044)
Supplement: Multimedia Appendix 2 [file medinform_v7i4e14044_app2.pdf]

Multimedia Appendix 2. Example of a patient complaining of muscle cramp

First, the patient completes DIAANA on the Web App. Below is an example where the patient is localizing his problem.

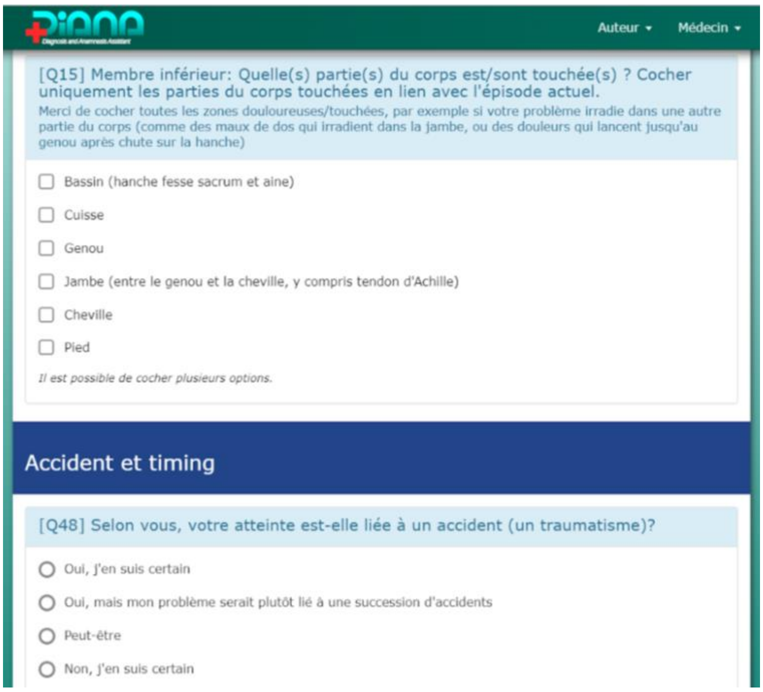

After selecting “jambe” (leg), the question “Q37” concerning the precise localization appears.

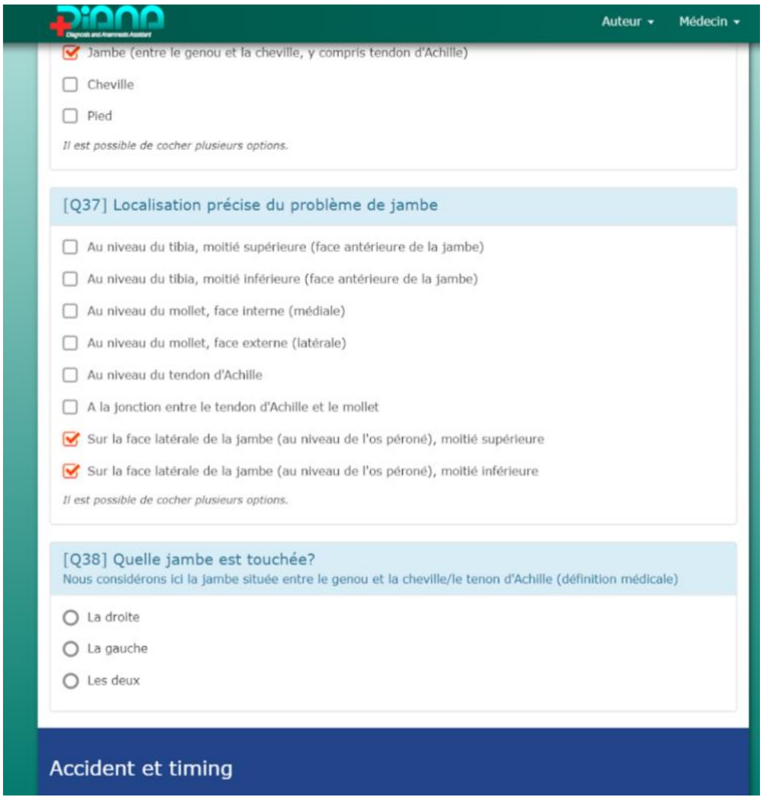

The patient is now describing a potential trauma, but in this case, the actual occurrence of trauma is unclear.

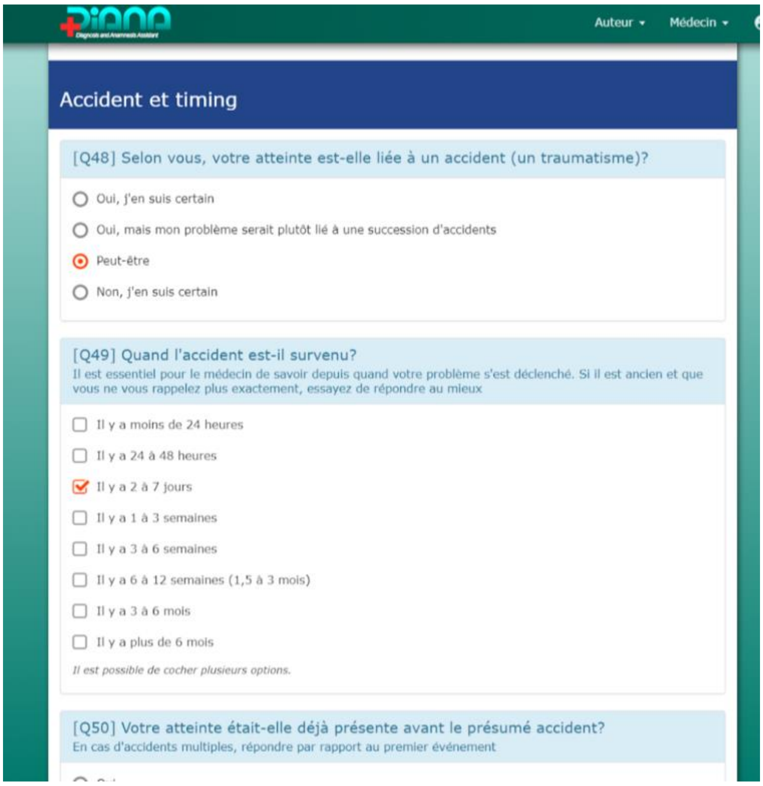

At the end of the form, the patient sends it to the medical team (button “Envoyer”).

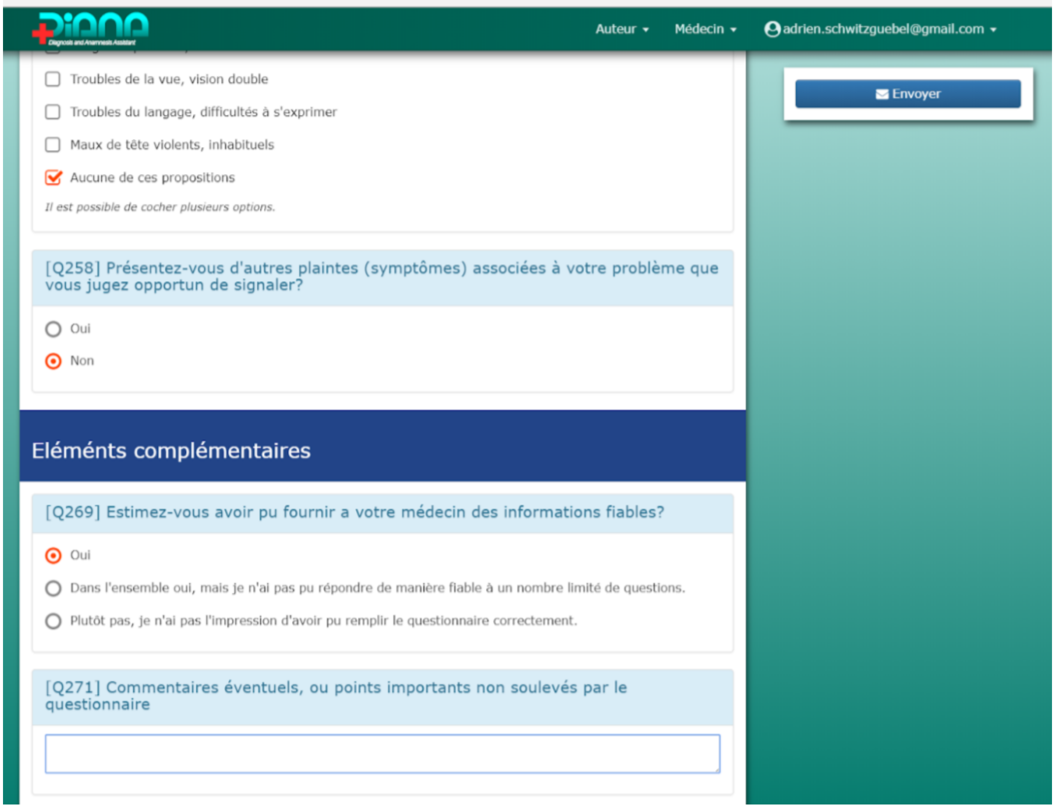

The physician can then access DIAANA's anamnesis summary …

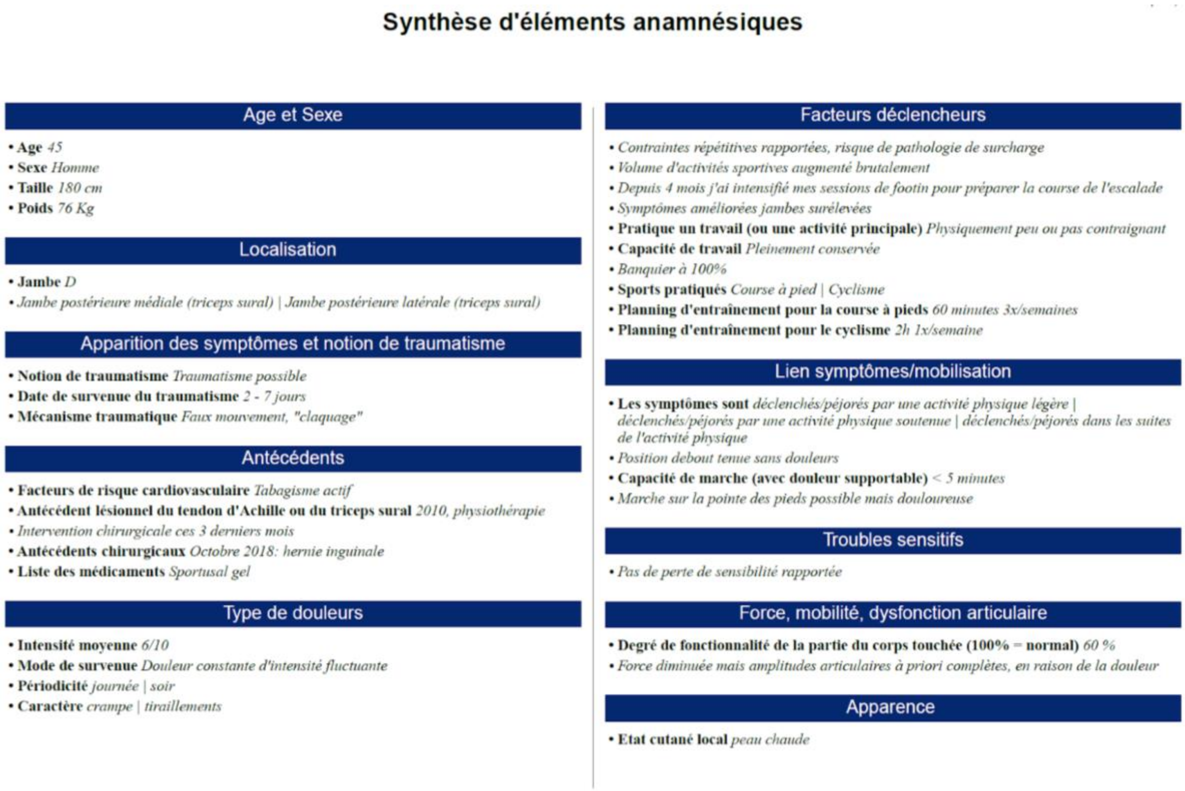

Synthèse d'éléments anamnésiques

Age et Sexe
- **Age** *45*
- **Sexe** *Homme*
- **Taille** *180 cm*
- **Poids** *76 Kg*

Localisation
- **Jambe** *D*
- *Jambe postérieure médiale (triceps sural) | Jambe postérieure latérale (triceps sural)*

Apparition des symptômes et notion de traumatisme
- **Notion de traumatisme** *Traumatisme possible*
- **Date de survenue du traumatisme** *2 - 7 jours*
- **Mécanisme traumatique** *Faux mouvement, "claquage"*

Antécédents
- **Facteurs de risque cardiovasculaire** *Tabagisme actif*
- **Antécédent lésionnel du tendon d'Achille ou du triceps sural** *2010, physiothérapie*
- *Intervention chirurgicale ces 3 derniers mois*
- **Antécédents chirurgicaux** *Octobre 2018: hernie inguinale*
- **Liste des médicaments** *Sportusal gel*

Type de douleurs
- **Intensité moyenne** *6/10*
- **Mode de survenue** *Douleur constante d'intensité fluctuante*
- **Périodicité** *journée | soir*
- **Caractère** *crampe | tiraillements*

Facteurs déclencheurs
- *Contraintes répétitives rapportées, risque de pathologie de surcharge*
- *Volume d'activités sportives augmenté brutalement*
- *Depuis 4 mois j'ai intensifié mes sessions de footin pour préparer la course de l'escalade*
- *Symptômes améliorées jambes surélevées*
- **Pratique un travail (ou une activité principale)** *Physiquement peu ou pas contraignant*
- **Capacité de travail** *Pleinement conservée*
- *Banquier à 100%*
- **Sports pratiqués** *Course à pied | Cyclisme*
- **Planning d'entraînement pour la course à pieds** *60 minutes 3x/semaines*
- **Planning d'entraînement pour le cyclisme** *2h 1x/semaine*

Lien symptômes/mobilisation
- **Les symptômes sont** *déclenchés/péjorés par une activité physique légère | déclenchés/péjorés par une activité physique soutenue | déclenchés/péjorés dans les suites de l'activité physique*
- *Position debout tenue sans douleurs*
- **Capacité de marche (avec douleur supportable)** *< 5 minutes*
- *Marche sur la pointe des pieds possible mais douloureuse*

Troubles sensitifs
- *Pas de perte de sensibilité rapportée*

Force, mobilité, dysfonction articulaire
- **Degré de fonctionnalité de la partie du corps touchée (100% = normal)** *60 %*
- *Force diminuée mais amplitudes articulaires à priori complètes, en raison de la douleur*

Apparence
- **Etat cutané local** *peau chaude*

… as well as the possible differential diagnoses list, with the corresponding emergency level for each condition.

Diagnostics différentiels - Résumé

(haute sensibilité, faible spécificité)

Diagnostics urgents

- Thrombose veineuse profonde

Diagnostics potentiellement urgents

- Lésion insertionnelle du gastrocnémien (tennis leg)
- Tableau clinique compatible avec un hématome ou une tuméfaction traumatique des tissus mous.

Diagnostics non-urgents

- Insuffisance veineuse des membres inférieurs
- Kyste de Baker
- Sciatalgie/cruralgie non déficitaire ± lombalgie
- Syndrome des loges chronique
- Tendinopathie des tendons médiaux de la cheville (ou: conflit osseux, lésion ostéochondrale, canal tarsien)
- Tuméfaction aspécifique des tissus mous (hématome, ganglion, tumeur, ...)

After a brief anamnesis and physical examination, the physician hesitates between the diagnosis of deep vein thrombosis and tennis leg. He can identify the potential risk factors that are present or absent when consulting the detailed information available on DIAANA for each diagnosis.

Diagnostics différentiels - Détails

(haute sensibilité, faible spécificité)

Diagnostics urgents

Thrombose veineuse profonde

Présence d'au moins un symptôme compatible avec une thrombose veineuse profonde

Si bilan nécessaire: utilité potentielle de l'écho-doppeler

Facteurs de risque

+ Tuméfaction, oedème
+ Type de douleur: crampe
+ Atteinte unilatérale
+ Chirurgie datant de 3 mois ou moins
+ Tabagisme (facteur de risque avéré en association avec contraception hormonale)
+ Localisation: jambe
- Type de douleur: brûlure ou froid douloureux
- Température, ou sudations
- Jambes lourdes
- >= 60 ans
- Immobilisation
- Notion d'antécédent de maladie thromboembolique
- Contraception hormonale
- Grossesse
- Tumeur ou cancer
- Localisation: arrière-pied
- Localisation: cheville
- Localisation: genou postérieur
- Localisation: cuisse
- Localisation: aine
- Localisation: méso-pied

Diagnostics potentiellement urgent

Lésion insertionnelle du gastrocnémien (tennis leg)

En cas de désinsertion importante du gastrocnémien, une ponction précoce d'hématome pourrait améliorer le pronostic.

Si bilan nécessaire: utilité potentielle de l'ultrasonographie

Facteurs de risque

+ Localisation: jambe postérieure
+ Zone(s) du corps touchée(s) (toutes) compatible(s) avec le diagnostic proposé
+ Mécanisme lésionnel: entorse, déchirure, claquage, faux mouvement
+ Traumatisme <= 6 semaines
+ Douleurs à la marche
+ Notion de surcharge ou stress mécanique répété excessif
- Craquement durant le traumatisme
- 31 - 40 ans
- Douleurs à la course
- Sport de pivot, ou avec sauts à répétition
- Potentiel problème de chaussage

Finally, the physician was able to order a Doppler ultrasound echo-Doppler with the help of DIAANA and the diagnosis of deep vein thrombosis was not mistaken for tennis leg.
