# Supplementary material for: Differential Diagnosis Assessment in Ambulatory Care With an Automated Medical History–Taking Device: Pilot Randomized Controlled Trial
Source: JMIR Med Inform. 2019 Nov 4;7(4):e14044. doi: 10.2196/14044 (PMC6913752; doi:10.2196/14044)
Supplement: Multimedia Appendix 3 [file medinform_v7i4e14044_app3.pdf]

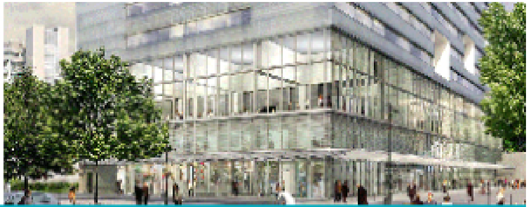

QUESTIONNAIRE DIAGNOSTICS DIFFERENTIELS

DMCPRU_Unité d'Urgences Ambulatoires

Merci de choisir au minimum 1 diagnostic différentiel et 5 au maximum.

← Précédent

Suivant →

Systèmes : neurologique central, organes internes, dermatologique

Couverture non exhaustive par DIANA

1. Diagnostics

- ☐ 1. Dermatose, maladie dermatologique (infection ou inflammation cutanée exclues)
- ☐ 2. Infection ou inflammation cutanée (dermo-hypodermite, érésipèle…)
- ☐ 3. Infection cutanée : localisation compatible avec abcès anal et kyste sacrococcygien
- ☐ 4. Traumatisme avec brèche cutanée
- ☐ 5. Médecine interne : atteinte neurologique centrale aigue (AVC, hémorragie...)
- ☐ 6. Médecine interne : atteinte neurologique centrale chronique (Parkinson, sclérose en plaque...)
- ☐ 7. Polyneuropathie dégénérative (diabétique, maladie des petites fibres…) ou autre neuropathie périphérique
- ☐ 8. Médecine interne : possible origine urogénitale
- ☐ 9. Médecine interne : possible origine des organes abdominaux (appendicite, calcul biliaire, gastrite…)
- ☐ 10. Traumatisme des organes abdominaux
- ☐ 11. Médecine interne : possible origine cardiaque ou aortique (infarctus, trouble du rythme, dissection…)
- ☐ 12. Médecine interne : possible origine pulmonaire (infection, asthme aigu, embolie, pneumothorax...)
- ☐ 13. Gynécologie : possible atteinte du sein
- ☐ 14. Maladie musculaire (myosite, myopathie…)

Membre supérieur

2. a) Diagnostics sans localisation segmentaire précise

- [ ] 1. Hématome ou tuméfaction traumatique des tissus mous (contusion, ecchymose…)
- [ ] 2. Atteinte musculaire ou tendineuse (claquage ou autre lésion musculaire, tendinopathies…)
- [ ] 3. Tuméfaction aspécifique non traumatique (lipome, kyste dermoïde…)
- [ ] 4. Ostéomyélite ou tumeur musculosquelletique
- [ ] 5. Fracture de stress
- [ ] 6. Nécrose aseptique
- [ ] 7. Syndrome des loges aigu
- [ ] 8. Syndrome des loges chronique
- [ ] 9. Douleurs constantes sous le plâtre
- [ ] 10. Pseudarthrose
- [ ] 11. (Péri-)arthrite inflammatoire (oligo- polyarthrite, spondylarthrite…)
- [ ] 12. Monoarthrite (septique ou rhumatismale: goutte, chondrocalcinose...)
- [ ] 13. Maladie musculaire (myosite, myopathie, …)
- [ ] 14. (Cervico-) brachialgies (radiculalgie du membre supérieur)
- [ ] 15. (Cervico-) brachialgies déficitaire
- [ ] 16. Algoneurodystrophie
- [ ] 17. Atteinte du nerf radial
- [ ] 18. Atteinte du nerf médian
- [ ] 19. Atteinte du nerf ulnaire
- [ ] 20. Atteinte du nerf musculocutanné
- [ ] 21. Atteinte du plexus brachial (plexopathie, Parsonnage-Turner…)
- [ ] 22. Syndrome du défilé thoracique
- [ ] 23. Thrombose veineuse profonde du membre supérieur
- [ ] 24. Polyneuropathie dégénérative (diabétique, maladie des petites fibres…), ou autre neuropathie périphérique

3. b) Epaule, ceinture scapulaire, bras

- [ ] 1. Fracture ou luxation de la clavicule
- [ ] 2. Traumatisme claviculaire d'évolution non favorable
- [ ] 3. Douleur originaire de l'articulation acromioclaviculaire
- [ ] 4. Tendinopathie ou déchirure du muscle pectoral
- [ ] 5. Fracture de l'humérus, Avulsion traumatique de la coiffe des rotateurs
- [ ] 6. Atteinte de la coiffe des rotateurs, conflit sous-acromial
- [ ] 7. Luxation gléno-humérale (luxation de l'épaule)
- [ ] 8. Instabilité gléno-humérale
- [ ] 9. Epaule gelée
- [ ] 10. Lésion du labrum de l'épaule
- [ ] 11. Atteinte dégénérative de l'épaule (arthrose ou autre)
- [ ] 12. Tendinopathie ou déchirure du biceps
- [ ] 13. Conflit d'épaule postérosupérieur
- [ ] 14. Atteinte du nerf axillaire
- [ ] 15. Atteinte du nerf suprascapulaire
- [ ] 16. Atteinte du nerf thoracique long

4. c) Coude, avant-bras

- [ ] 1. Atteinte du biceps brachial distal ou de son enthèse
- [ ] 2. Epicondylite latérale ou médiale
- [ ] 3. Atteinte du ligament collatéral latéral du coude
- [ ] 4. Atteinte du ligament collatéral médial du coude
- [ ] 5. Luxation du coude
- [ ] 6. Atteinte du triceps brachial distal ou de son enthèse
- [ ] 7. Bursite oléchranienne non septique
- [ ] 8. Bursite septique
- [ ] 9. Atteinte articulaire du coude (arthrose, nodule ostéochondromateux, conflit…)
- [ ] 10. Fracture du coude
- [ ] 11. Atteinte du nerf musculocutané

Rachis et cage thoracique

5. Diagnostics

- ☐ 1. Hématome ou tuméfaction traumatique des tissus mous (contusion, ecchymose…)
- ☐ 2. Tuméfaction aspécifique non traumatique (lipome, kyste dermoïde…)
- ☐ 3. Maladie musculaire (myosite, myopathie…)
- ☐ 4. (Péri-)arthrite inflammatoire (oligo- polyarthrite, spondylarthrite…)
- ☐ 5. Rachialgies aspécifiques (cervico- dorso- scapulo- lombalgies ou douleurs de la musculature paravertébrale)
- ☐ 6. Rachialgies avec facteur de gravité (tassement, spondylodiscite, fracture traumatique…)
- ☐ 7. (Lombo)-sciatalgies/cruralgies (sur hernie discale par exemple)
- ☐ 8. (Lombo)-sciatalgies /cruralgies déficitaires
- ☐ 9. Dysfonction articulaire sacro-iliaque mécanique
- ☐ 10. Pyriformalgie
- ☐ 11. (Cervico-)brachialgies (radiculalgie du membre supérieur)
- ☐ 12. (Cervico-)brachialgies déficitaire
- ☐ 13. Canal lombaire étroit
- ☐ 14. Spondylolisthésis
- ☐ 15. Fracture de la cage thoracique
- ☐ 16. Cage thoracique douloureuse : syndrome de Cyriax, syndrome de Tietze, atteinte musculaire intercostale
- ☐ 17. Tendinopathie ou déchirure du muscle pectoral

Membre inférieur

6. a) Diagnostics

- [ ] 1. Hématome ou tuméfaction traumatique des tissus mous (contusion, ecchymose...)
- [ ] 2. Atteinte musculaire ou tendineuse (claquage ou autre lésion musculaire, tendinopathies...)
- [ ] 3. Tuméfaction aspécifique non traumatique (lipome, kyste dermoïde...)
- [ ] 4. Ostéomyélite ou tumeur musculosquelletique
- [ ] 5. Fracture de stress
- [ ] 6. Nécrose aseptique
- [ ] 7. Syndrome des loges aigu
- [ ] 8. Syndrome des loges chronique
- [ ] 9. Douleurs constantes sous le plâtre
- [ ] 10. Pseudarthrose
- [ ] 11. (Péri-)arthrite inflammatoire (oligo- polyarthrite goutteuse, spondylarthrite...)
- [ ] 12. Monoarthrite (septique ou rhumatismale: goutte, chondrocalcinose...)
- [ ] 13. Maladie musculaire (myosite, myopathie...)
- [ ] 14. (Lombo)-sciatalgies/cruralgies (sur hernie discale par exemple)
- [ ] 15. (Lombo)-sciatalgies /cruralgies déficitaires
- [ ] 16. Algoneurodystrophie
- [ ] 17. Atteinte du nerf fémoral
- [ ] 18. Atteinte du nerf sciatique
- [ ] 19. Atteinte du nerf saphène (y compris branches géniculées)
- [ ] 20. Atteinte du nerf fibulaire (superficiel ou profond)
- [ ] 21. Atteinte du nerf sural
- [ ] 22. Polyneuropathie dégénérative (diabétique, maladie des petites fibres...), ou autre neuropathie périphérique
- [ ] 23. Syndrome des jambes sans repos
- [ ] 24. Insuffisance artérielle
- [ ] 25. Insuffisance artérielle avec critères d'urgence
- [ ] 26. Insuffisance veineuse des membres inférieurs
- [ ] 27. Thrombose veineuse profonde du membre inférieur

7. b) Anneau pelvien

- [ ] 1. Pubalgie liée à la paroi abdominale (hernie, tendinite ou déchirure des muscles abdominaux)
- [ ] 2. Tendinopathie ou déchirure des adducteurs
- [ ] 3. Tendinopathie ou déchirure du muscle iliopsoas
- [ ] 4. Fracture de la hanche
- [ ] 5. Atteinte mécanique de l'articulation de la hanche (coxarthrose ou lésion chondrale, impingement, atteinte labrum)
- [ ] 6. Syndrome douloureux du grand trochanter (déchirure ou tendinopathie du moyen fessier...)
- [ ] 7. Arrachement apophysaire pelvien (arrachement de l'insertion d'un muscle au niveau du bassin)
- [ ] 8. Dysfonction articulaire sacro-iliaque d'origine mécanique
- [ ] 9. Pyriformalgies
- [ ] 10. Atteinte du nerf pudental (ou des nerfs ilio-inguinal, ilio-hypogastrique, obturateur)

8. c) Cuisse, genou, jambe

- [ ] 1. Lésion aigue de l'appareil extenseur
- [ ] 2. Fracture diaphysaire (fémur ou tibia-péronné)
- [ ] 3. Déchirure ou atteinte des muscles ischios jambiers
- [ ] 4. Syndrome de la bandelette ilio-tibiale
- [ ] 5. Fracture du genou ou luxation fémoro-tibiale
- [ ] 6. Entorse sévère du genou (ligaments croisés, impact osseux)
- [ ] 7. Entorse isolée d'un ligament collatéral du genou (généralement dite « légère »)
- [ ] 8. Luxation patellaire
- [ ] 9. Atteinte méniscale (traumatique ou dégénératif)
- [ ] 10. Lésion cartilagineuse du genou traumatique aigue
- [ ] 11. Lésion cartilagineuse du genou chronique, gonarthrose
- [ ] 12. Kyste de Baker
- [ ] 13. Tendinopathie de la patte d'oie
- [ ] 14. Tendinopathie fémoropatellaire ou syndrome fémoropatellaire
- [ ] 15. Bursite (patellaire) non septique
- [ ] 16. Bursite (patellaire) septique
- [ ] 17. Tennis leg (déchirure du muscle gastrocnémien)
- [ ] 18. Périostite ou fracture de stress tibiale
- [ ] 19. Méralgie paresthétique (neuropathie du nerf cutané fémoral latéral)

9. d) Cheville et pied

- 1. Fracture de la cheville ou du pied (malléole, arrière-pied, avant-pied…)
- 2. Traumatisme de cheville (>3 semaines) d'évolution défavorable (lésion chondrale ou autre lésion passée inaperçue, synovite réactionnelle, rééducation inadéquate)
- 3. Entorse de cheville avec suspicion d'atteinte isolée des ligaments latéraux
- 4. Entorse de cheville avec suspicion d'atteinte de la syndesmose
- 5. Rupture du tendon d'Achille
- 6. Tendinopathie achilléenne
- 7. Tendinopathie ou déchirure des péroniers
- 8. Tendinopathie ou déchirure des tendons médiaux de la cheville (tibial postérieur)
- 9. Entorse de Lisfranc ou de Chopard
- 10. Fasciite plantaire
- 11. Entorse de l'hallux (Turf toe)
- 12. Métatharsalgies (Morton, plaque palmaire, bourses, sésamoïdes…)
- 13. Déformation des orteils (hallux valgus, orteils en griffe…)
- 14. Syndrome du canal tarsien (ou du nerf tibial)
- 15. Arthrose
- 16. Luxation articulaire
