# Supplementary material for: Differential Diagnosis Assessment in Ambulatory Care With an Automated Medical History–Taking Device: Pilot Randomized Controlled Trial
Source: JMIR Med Inform. 2019 Nov 4;7(4):e14044. doi: 10.2196/14044 (PMC6913752; doi:10.2196/14044)
Supplement: Multimedia Appendix 4 [file medinform_v7i4e14044_app4.pdf]

Multimedia Appendix 4. Type of differential diagnoses selected by the senior physician for each level of complexity.

	**AMHTD**	**Control group**
Low complexity<br>(1-2 DD to find)	Soft tissue swelling (4)<br>Muscle or tendon sprain (2)<br>Shoulder fracture<br>Shoulder osteoarthritis<br>Triceps brachialis tendinosis<br>Olecranon bursitis<br>Aspecific back pain (2)<br>Inflammatory (peri)arthritis<br>Patellar bursitis<br>Septic patellar bursitis<br>Ankle fracture (2)<br>Poor evolution ankle sprain<br>Ankle sprain (3)	Soft tissue swelling (1)<br>Muscle or tendon sprain (1)<br>Dermatosis (2)<br>Dermohypodermitis (2)<br>Aspecific back pain (2)<br>(Lombo-)sciatica<br>Inflammatory (peri)arthritis<br>Ankle fracture (3)<br>Ankle sprain (3)<br>Ankle osteoarthritis
Moderate complexity<br>(3 DD to find)	Soft tissue swelling (2)<br>Dermohypodermitis<br>(Cervico-)brachalgia<br>(Cervico-)brachalgia & weakness<br>Aspecific back pain (2)<br>Back pain with red flag<br>Inflammatory (peri-)arthritis<br>Monoarthritis<br>Ankle fracture<br>Ankle sprain<br>Lisfranc or Chopard sprain (2)<br>Ankle osteoarthritis	Soft tissue swelling (2)<br>Central neurologic disease<br>Myositis (2)<br>Collarbone fracture or luxation<br>Shoulder fracture<br>Glenohumeral luxation<br>Posterosuperior shoulder impingement<br>Aspecific back pain (3)<br>Back pain with red flag (2)<br>(Lombo-)sciatalgia (2)<br>(Lombo-)sciatalgia & weakness<br>Monoarthritis<br>Leg extensors strain<br>Knee fracture<br>Knee collateral ligament sprain<br>Meniscal tear<br>Knee cartilaginous traumatic lesion<br>Knee osteoarthritis (2)<br>Baker cyst<br>Septic patellar bursitis<br>Ankle fracture (3)<br>Ankle sprain (3)<br>Tibiofibular syndesmosis sprain<br>Peroneous tendons tendinosis<br>Lisfranc or Chopard sprain

	**AMHTD**	**Control group**
High complexity<br>(4-5 DD to find	Soft tissue swelling (4)<br>Muscle or tendon sprain (7)<br>Inflammatory (peri-)arthritis (2)<br>Monoarthritis (4)<br>Myositis<br>Polyneuropathy<br>Arterial insufficiency<br>Deep vein thrombosis<br>Stress fracture (2)<br>Rotator cuff lesion<br>Frozen shoulder<br>Shoulder osteoarthritis<br>Aspecific back pain<br>Back pain with red flag<br>(Lombo-)sciatalgia<br>Abdominal wall originated pubalgia<br>Adductor sprain (2)<br>Hip: osteoarthritis, impingement, labral tear<br>Greater trochanteric pain syndrome<br>Leg extensors strain<br>Long bone fracture<br>Knee fracture (2)<br>Severe knee sprain<br>Knee collateral ligament sprain<br>Patellar luxation<br>Meniscal tear (4)<br>Knee osteoarthritis (3)<br>Baker cyst<br>Patellar bursitis (2)<br>Ankle fracture<br>Ankle sprain	Soft tissue swelling (3)<br>Muscle or tendon sprain (2)<br>Stress fracture (2)<br>Inflammatory (peri-)arthritis<br>Monoarthritis<br>Median nerve neuropathy<br>Collarbone fracture or luxation<br>Shoulder fracture<br>Shoulder luxation<br>(Lombo-)sciatica<br>(Lombo-)sciatalgia & weakness<br>Femoral nerve neuropathy<br>Hip: osteoarthritis, impingement, labral tear<br>Greater trochanteric pain syndrome<br>Leg extensors strain<br>Severe knee sprain (2)<br>Knee collateral ligament sprain<br>Patellar luxation<br>Meniscal tear (2)<br>Knee cartilaginous traumatic lesion<br>Patellar tendinopathy<br>Tibial periostitis or stress fracture<br>Ankle fracture<br>Ankle sprain<br>Tibiofibular syndesmosis sprain<br>Peroneous tendons tendinosis<br>Tibialis posterior tendinosis<br>Lisfranc or Chopard sprain<br>Metatarsalgia
