# Supplementary material for: Differential Diagnosis Assessment in Ambulatory Care With an Automated Medical History–Taking Device: Pilot Randomized Controlled Trial
Source: JMIR Med Inform. 2019 Nov 4;7(4):e14044. doi: 10.2196/14044 (PMC6913752; doi:10.2196/14044)
Supplement: Multimedia Appendix 5 [file medinform_v7i4e14044_app5.pdf]

Multimedia Appendix 5. Differential diagnoses found by the AMHTD.

	Mean ± SD (range)
**AMHTD-suggested DD (%)**	
DD accuracy	73 ± 30 (0-100)
Low complexity index (1-2 DDs to find)	91 ± 20 (50-100)
Moderate complexity index (3 DDs to find)	67 ± 24 (33-100)
High complexity index (4-5 DDs to find)	59 ± 32 (0-100)
**Potential bias induced by the DDs**	
AMHTD superfluous DDs (n)	5 ± 4 (0-12)
Correct DDs not followed by residents (%)	10 ± 19 (0-50)
Incorrect DDs followed by residents (%)	21 ± 51 (0-250)

DD: differential diagnoses.
