# Supplementary material for: Differential Diagnosis Assessment in Ambulatory Care With an Automated Medical History–Taking Device: Pilot Randomized Controlled Trial
Source: JMIR Med Inform. 2019 Nov 4;7(4):e14044. doi: 10.2196/14044 (PMC6913752; doi:10.2196/14044)
Supplement: Multimedia Appendix 6 [file medinform_v7i4e14044_app6.pdf]

Multimedia Appendix 6. Automated medical history-taking device group: patient and resident physician satisfaction.

	Mean ± SD (range)
**Patient feedback**	
Overall satisfaction	4.3 ± 1 (2-5)
Understandability of AMHTD questions	4.4 ± 1 (4-5)
Symptomatology accurately described (%)	26/29 (90)
Wish to keep summary at home (%)	12/29 (41)
Wish to use the AMHTD at home (%)	14/29 (48)
**Physician feedback**	
Wish to obtain the integrality of the AMHTD summary form (%)	20/29 (69)
AMHTD helps finding DDs (%)	8/29 (28)
AMHTD allows to gain time	3.1 ± 1 (1-4)

AMHTD**:** automated medical history-taking devices; DD: differential diagnoses.
